# 687. Impact of a Risk-Stratified Febrile Neutropenia Guideline on Antibiotic Utilization at a Free-Standing Children’s Hospital

**DOI:** 10.1093/ofid/ofaf695.226

**Published:** 2026-01-11

**Authors:** Leila C Posch, Teresa Rushing, Darra Drucker, Priya Edward, Etan Orgel, Regina Orbach

**Affiliations:** Children's Hospital Los Angeles, Los Angeles, California; Children's Hospital Los Angeles, Los Angeles, California; Children's Hospital Los Angeles, Los Angeles, California; Children's Hospital Los Angeles, Los Angeles, California; Children's Hospital Los Angeles, Keck School of Medicine of USC, Los Angeles, California; Children Hospital Los Angeles, los angeles, CA

## Abstract

**Background:**

Febrile neutropenia (FN) is a common complication of pediatric cancer treatment, and broad-spectrum empiric antibiotic therapy (EAT) improves outcomes. However, high concern for infection can lead to unnecessarily broad antibiotic exposure, which is associated with increased adverse effects and antimicrobial resistance. National guidelines recommend a risk-stratified approach to EAT in pediatric cancer patients guided by local epidemiology. In 2023, Children’s Hospital Los Angeles (CHLA) updated the FN guideline to (1) address prior universal empiric meropenem use for FN, and (2) reduce overall antibiotic utilization rates (AUR) via earlier de-escalation. This study evaluated the impact of this quality improvement (QI) intervention on AUR and patient outcomes.

**Figure:**
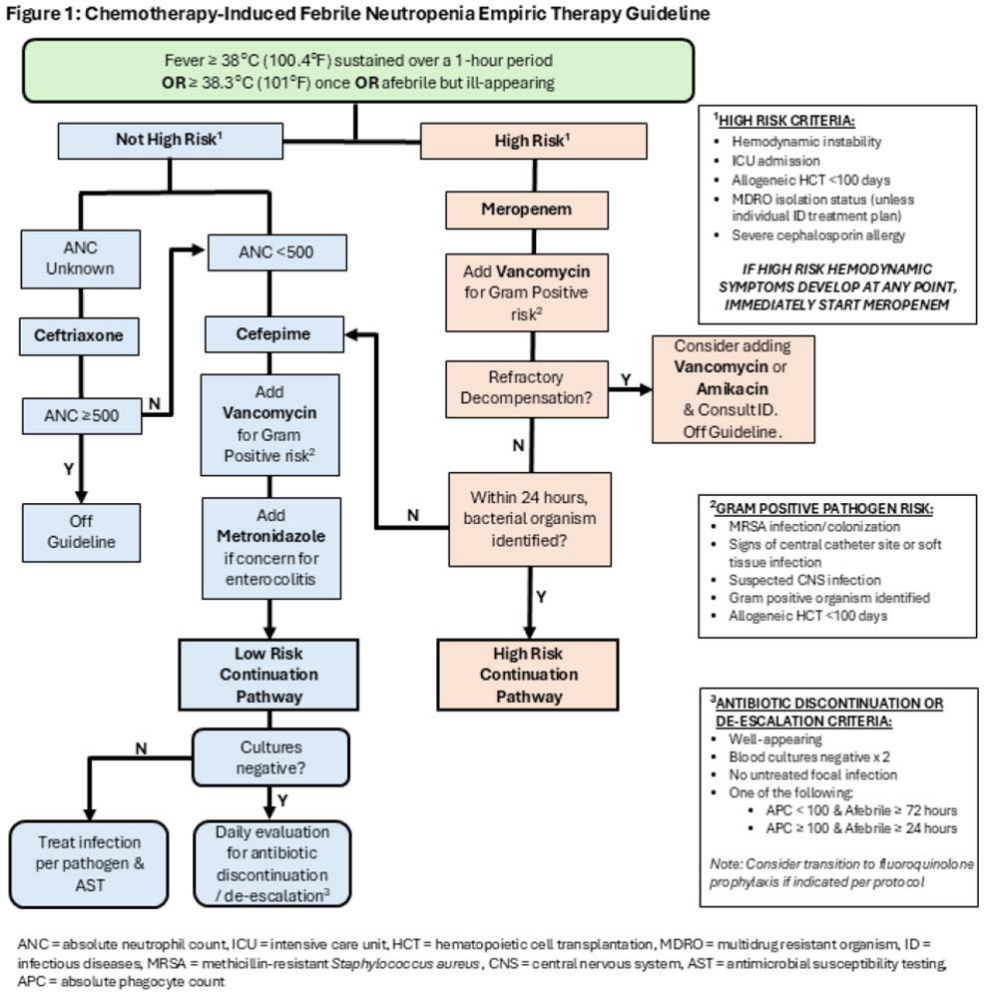

**Figure:**
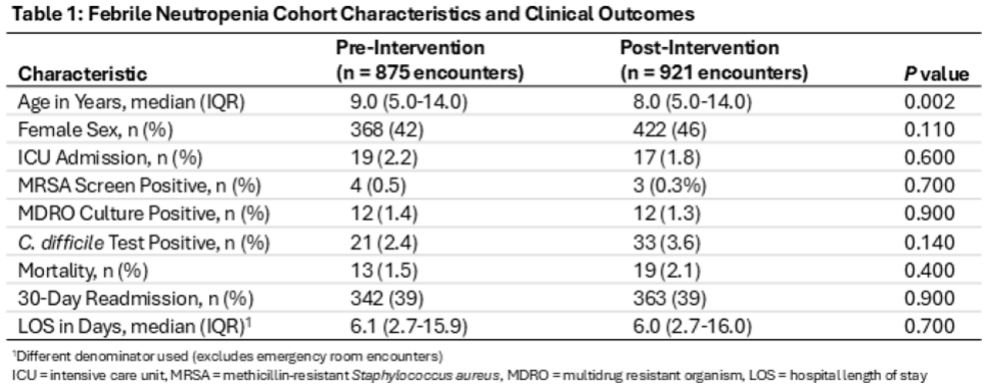

**Methods:**

Extracted data were compared for patient encounters receiving meropenem, cefepime, vancomycin, or ceftriaxone for FN indication in the pre- (Nov 2022-Oct 2023) and post-intervention (Nov 2023-Oct 2024) periods. The FN guideline was implemented 11/1/2023 (Figure 1). Patients without a malignancy or hematopoietic cell transplantation (HCT) diagnosis were excluded. The primary outcome was hospital-wide AUR as days of therapy per 1000 days present. Secondary outcomes were intensive care unit stay, mortality, readmission, hospital length of stay, and microbiologic results.

**Figure:**
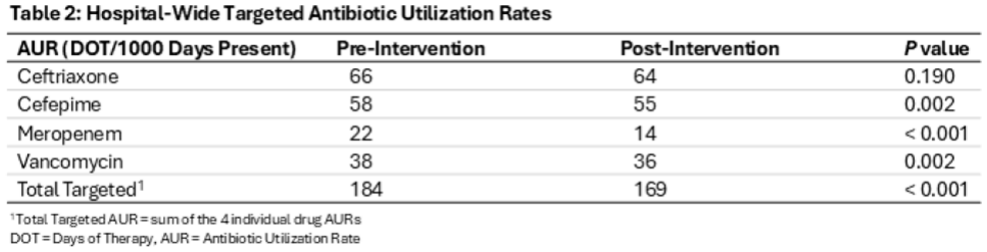

**Results:**

875 patient encounters in the pre- and 921 in the post-intervention period received > = 1 antibiotic for FN. Baseline characteristics were similar between groups, except median age (9 vs 8 years) (Table 1). Post-intervention, significant decreases were found in hospital-wide total AUR (184 vs 169, p < 0.001) and meropenem AUR (22 vs 14, p < 0.001). Vancomycin and cefepime AUR also decreased (Table 2). There were no differences in secondary outcomes (Table 1).

**Conclusion:**

Implementation of a risk-stratified pediatric FN guideline with de-escalation criteria led to significant reduction in hospital-wide antibiotic utilization, particularly for meropenem, without adversely impacting clinical outcomes for FN patients. Limitations include potential concurrent unrelated interventions influencing hospital AUR. Future studies will assess guideline impact on FN population AUR and specific barriers to guideline adherence.

**Disclosures:**

All Authors: No reported disclosures

